# Comparison of the Characteristics, Management, and Outcomes of STEMI Patients Presenting With vs. Those of Patients Presenting Without COVID-19 Infection: A Systematic Review and Meta-Analysis

**DOI:** 10.3389/fcvm.2022.831143

**Published:** 2022-03-14

**Authors:** Yanjiao Wang, Linlin Kang, Ching-Wen Chien, Jiawen Xu, Peng You, Sizhong Xing, Tao-Hsin Tung

**Affiliations:** ^1^Shenzhen Bao'an District Traditional Chinese Medicine Hospital, Shenzhen, China; ^2^Institute for Hospital Management, Tsing Hua University, Shenzhen, China; ^3^Evidence-Based Medicine Center, Taizhou Hospital of Zhejiang Province Affiliated to Wenzhou Medical University, Linhai, China

**Keywords:** COVID-19, SARS-CoV-2, mortality, ST-segment elevation myocardial infarction, STEMI

## Abstract

**Objectives:**

This study aimed to investigate the differences in the characteristics, management, and clinical outcomes of patients with and that of those without coronavirus disease 2019 (COVID-19) infection who had ST-segment elevation myocardial infarction (STEMI).

**Methods:**

Databases including Web of Science, PubMed, Cochrane Library, and Embase were searched up to July 2021. Observational studies that reported on the characteristics, management, or clinical outcomes and those published as full-text articles were included. The Newcastle-Ottawa Scale (NOS) was used to assess the quality of all included studies.

**Results:**

A total of 27,742 patients from 13 studies were included in this meta-analysis. Significant delay in symptom onset to first medical contact (SO-to-FMC) time (mean difference = 23.42 min; 95% CI: 5.85–40.99 min; *p* = 0.009) and door-to-balloon (D2B) time (mean difference = 12.27 min; 95% CI: 5.77–18.78 min; *p* = 0.0002) was observed in COVID-19 patients. Compared to COVID-19 negative patients, those who are positive patients had significantly higher levels of C-reactive protein, D-dimer, and thrombus grade (*p* < 0.05) and showed more frequent use of thrombus aspiration and glycoprotein IIbIIIa (Gp2b3a) inhibitor (*p* < 0.05). COVID-19 positive patients also had higher rates of in-hospital mortality (OR = 5.98, 95% CI: 4.78–7.48, *p* < 0.0001), cardiogenic shock (OR = 2.75, 95% CI: 2.02–3.76, *p* < 0.0001), and stent thrombosis (OR = 5.65, 95% CI: 2.41–13.23, *p* < 0.0001). They were also more likely to be admitted to the intensive care unit (ICU) (OR = 4.26, 95% CI: 2.51–7.22, *p* < 0.0001) and had a longer length of stay (mean difference = 4.63 days; 95% CI: 2.56–6.69 days; *p* < 0.0001).

**Conclusions:**

This study revealed that COVID-19 infection had an impact on the time of initial medical intervention for patients with STEMI after symptom onset and showed that COVID-19 patients with STEMI were more likely to have thrombosis and had poorer outcomes.

## Introduction

An eventual pandemic brought by the coronavirus disease 2019 (COVID-19) caused by severe acute respiratory syndrome coronavirus 2 (SARS-CoV-2) resulted in plenty of deaths and has had a strong impact on the world's healthcare system (1–3). Although the disease is predominantly characterized by respiratory symptoms, including pneumonia, dyspnea, and cough (4), various extrapulmonary features, such as myocardial damage, arrhythmia, thrombotic events, and renal injury have also been observed (5, 6).

A type of heart attack called ST-segment elevation myocardial infarction (STEMI) is usually caused by thrombotic occlusion at the site of a ruptured plaque in the coronary artery (7). Although the survival rates of STEMI patients have improved, it is still associated with high morbidity and mortality worldwide with a 1-year mortality rate of up to 10% (8–10). The COVID-19 pandemic may lead to a decrease in the number of STEMI admissions and could have a significant impact on the reperfusion strategy for patients with STEMI (11, 12). The tendency of patients with COVID-19 to be predisposed to cardiac arrest and coronary thrombosis due to increased inflammation, platelet activation, endothelial dysfunction, and SARS-CoV-2 invasion of cardiomyocytes has been reported (13–15). Moreover, data regarding the characteristics, management strategies, and clinical outcomes including in-hospital mortality and cardiogenic shock in patients presenting with STEMI concurrent with COVID-19 infection are limited (16). Accordingly, we aimed to conduct a systematic review and meta-analysis to compare the characteristics, management, and clinical outcomes between the COVID-19 and non-COVID-19 patients concomitant STEMI.

## Methods

### Literature Search

We performed a literature search using databases including Web of Science (Beijing), PubMed (Bethesda), Cochrane Library (UK), and Embase (Amsterdam) for relevant papers without language limitation on July 31, 2021. The search strategy included a mix of MeSH and free-text terms relevant to the critical concept of “STEMI” and “COVID-19” (Table 1). The protocol for this meta-analysis was registered at PROSPERO under the number CRD42021283880.

**Table 1:** Search strategy.

**Database**		**Searching key words**
PubMed	(1) “ST Segment Elevation Myocardial Infarction”: 9451	(10) SARS-CoV-2: 106826
	(2) “ST Elevated Myocardial Infarction”: 317	(11) “Coronavirus disease 19”: 1603
	(3) STEMI: 28060	(12) “Severe Acute Respiratory Syndrome Coronavirus 2”: 16865
	(4) “Acute myocardial infarction”: 61630	(13) “novel coronavirus”: 9766
	(5) AMI: 25165	(14) “2019 novel coronavirus”: 1550
	(6) “Acute coronary syndromes”: 13188	(15) #1 or #2 or #3 or #4 or #5 or #6 or #7: 208085
	(7) ACS: 116546	(16) #8 or #9 or #10 or #11 or #12 or #13 or #14: 169136
	(8) “SARSCoV-2 pandemic”: 120	(17) #15 and #16: 1340
	(9) COVID-19: 168784	
Web of science	(1) “ST Segment Elevation Myocardial Infarction”: 17531	(10) SARS-CoV-2: 127748
	(2) “ST Elevated Myocardial Infarction”: 1899	(11) “Coronavirus disease 19”: 3460
	(3) STEMI: 23388	(12) “Severe Acute Respiratory Syndrome Coronavirus 2”: 58794
	(4) “Acute myocardial infarction”: 145384	(13) “novel coronavirus”: 14678
	(5) AMI: 44201	(14) “2019 novel coronavirus”: 2224
	(6) “Acute coronary syndromes”: 27560	(15) #1 or #2 or #3 or #4 or #5 or #6 or #7: 248982
	(7) ACS: 58425	(16) #8 or #9 or #10 or #11 or #12 or #13 or #14: 262441
	(8) “SARSCoV-2 pandemic”: 25	(17) #15 and #16: 1098
	(9) COVID-19: 248069	
Cochrane library	(1) “ST Segment Elevation Myocardial Infarction”: 4031	(10) SARS-CoV-2: 322
	(2) “ST Elevated Myocardial Infarction”: 156	(11) “Coronavirus disease 19”: 43
	(3) STEMI: 3616	(12) “Severe Acute Respiratory Syndrome Coronavirus 2”: 631
	(4) “Acute myocardial infarction”: 9325	(13) “novel coronavirus”: 497
	(5) AMI: 3603	(14) “2019 novel coronavirus”: 55
	(6) “Acute coronary syndromes”: 2562	(15) #1 or #2 or #3 or #4 or #5 or #6 or #7: 19050
	(7) ACS: 4853	(16) #8 or #9 or #10 or #11 or #12 or #13 or #14: 6784
	(8) “SARSCoV-2 pandemic”: 52	(17) #15 and #16: 31
	(9) COVID-19: 6666	
Embase	('acute myocardial infarction':ti,ab,kw OR ami:ti,ab,kw OR 'acute coronary syndromes':ti,ab,kw OR acs:ti,ab,kw OR 'st segment elevation myocardial infarction':ti,ab,kw OR 'st elevated myocardial infarction':ti,ab,kw OR stemi:ti,ab,kw) AND ('sarscov-2 pandemicor COVID-19':ti,ab,kw OR 'sars cov 2':ti,ab,kw OR 'coronavirus disease 19':ti,ab,kw OR 'novel coronavirus':ti,ab, kw OR 'severe acute respiratory syndrome coronavirus 2':ti,ab,kw) AND [1-1-1900]/sd NOT [1-8-2021]/sd; result = 233	

### Study Selection

Studies were included if they met the following inclusion criteria: (i) studies involving STEMI patients; (ii) the exposure group included patients diagnosed with COVID-19 using PCR test or had a high index of clinical suspicion, and the control group included patients without COVID-19; (iii) studies that reported at least one of the following information: characteristics, management strategy, or clinical outcomes; (iv) relevant cohort studies, cross-sectional studies, case series, and case-control studies. Two independent authors screened the titles and abstracts of all relevant studies and identified whether they met the inclusion criteria by reviewing the full text of each potential study. Any discrepancy was resolved through consensus with a third author.

### Data Extraction and Quality Assessment

Relevant data from all included studies were extracted by two authors independently, and any disagreement was resolved by discussion with a third author. The following data were extracted: authors, publication year, country, study design, study subject, sample size, mean age of patients/subjects, sex, comparison period, participant characteristics, management strategies, and clinical outcomes. The Newcastle–Ottawa Scale (NOS), which includes participant selection, comparability, and outcome, was used to assess the quality of the included studies. Likewise, all included studies were rated by two authors independently, and any discrepancy was adjudicated by consensus.

### Statistical Analysis

We used Review Manager 5.4 (The Nordic Cochrane Center, Cochrane Collaboration, 2020, Denmark) to perform the statistical analysis. If studies only reported median values and interquartile ranges (IQR), means and *SD*s were calculated according to the Box-Cox method (17). Categorical variables were presented as odds ratios (ORs), including 95% CIs, and continuous variables were presented as the mean difference (MD) or standardized mean difference (SMD), including 95% CI. Heterogeneity was assessed using the I^2^ statistic and the *p*-value of the chi-square test. The I^2^ statistic > 50% indicates significant heterogeneity. The choice between the fixed and random effects models depended on the comparability among the studies. A two-tailed *p*-value of <0.05 was interpreted to be statistically significant. The risk of publication bias was evaluated using the funnel plots.

## Results

### Characteristics of Included Studies

**Figure 1:**
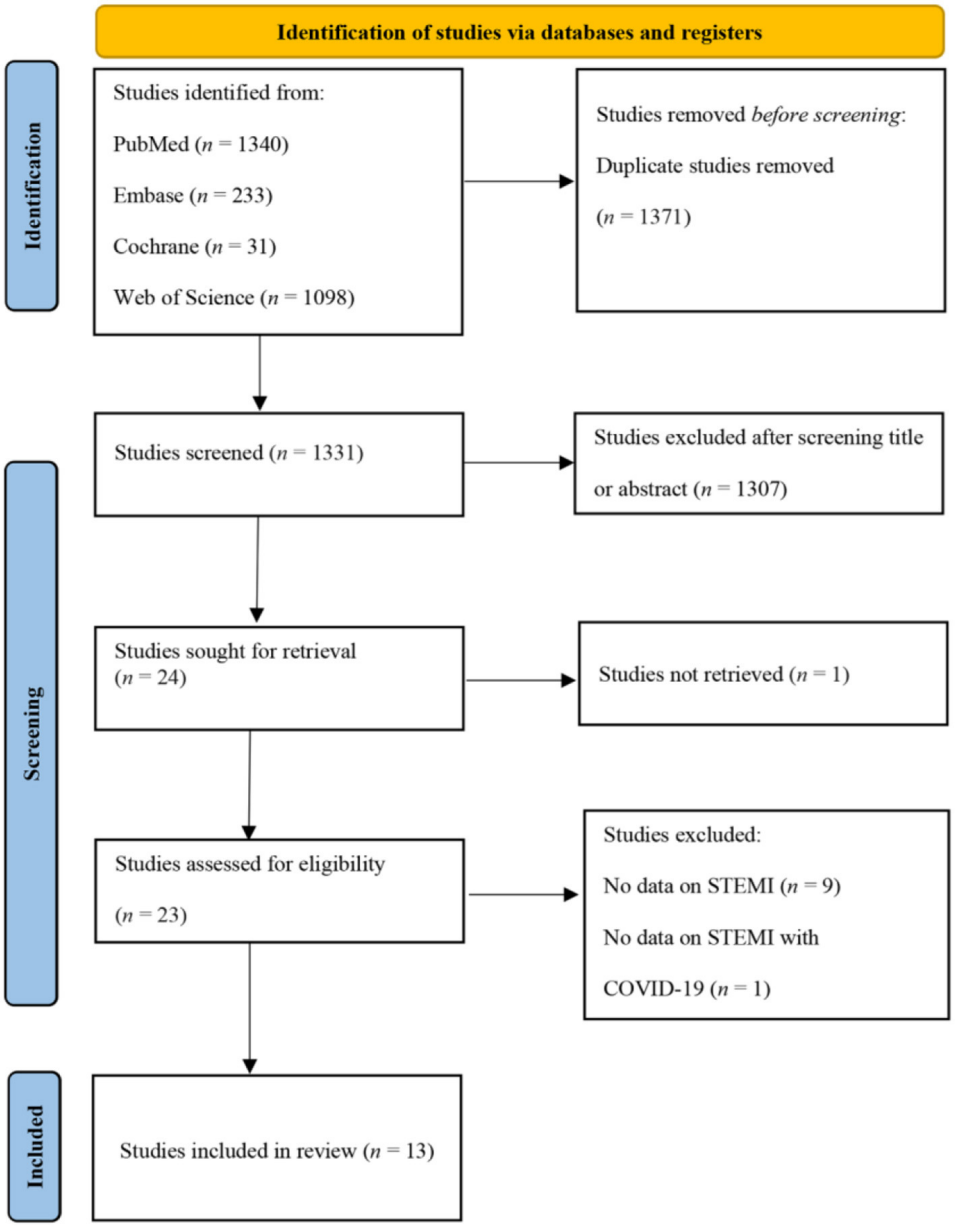
PRISMA flow diagram.

A total of 2,702 articles were retrieved through electronic database searches, of which 1,371 were duplicates. After screening the titles and abstracts, 24 potential articles were assessed for eligibility after a full-text review, and 13 articles (18–30) with a total of 27,742 patients were finally included (Figure 1). A summary of the main characteristics of these 13 studies and the baseline characteristics of all study subjects is presented in Tables 2A,B. One study originated from Poland (19), two each from the United Kingdom (24, 28), France (18, 21), Turkey (20, 30), Italy (25, 26), and Spain (27, 29), and the remaining two studies (22, 23) were international studies. The NOS score for all included studies varied from 5 to 8 points.

**Table 2A:** Characteristics of included studies.

**References**	**Country**	**Study design**	**Study group**	**Participants characteristics**	**Comparison period**	**COVID-19 diagnosis approach/time to diagnosis**	**Major findings**
Popovic et al. (18)	France	Monocentric cohort study	COVID-19 STEMI	*n* = 11, age 63.6 ± 17.4 years, 63.9% males	26/2/2020–10/5/2020	RT-PCR or typical clinical features plus CT results/NA	D2B time, Laboratory values, Primary angioplasty, MINOCA, Stent implantation, Gp2b3a inhibitor use, TIMI status, In-hospital mortality
			Non-COVID-19 STEMI	*n* = 72, age 62.5 ± 12.6 years, 73.6% males	26/2/2020–10/5/2020		
Siudak et al. (19)	Poland	Multicentric cohort study	COVID-19 STEMI	*n* = 145, age 63.19 ± 12.55 years, 71.33% males	13/3/2020–13/5/2020	Swabs for molecular RT-PCR testing/NA	SO-to-FMC time
			Non-COVID-19 STEMI	*n* = 2276, age 65.43 ± 12.23 years, 67.65% males	13/3/2020–13/5/2020		
Kiris et al. (20)	Turkey	Multicentric cross-sectional study	COVID-19 STEMI	*n* = 65, age 66.8 ± 12.0 years, 68% males	11/3/2020–15/5/2020	Nasal/pharyngeal swabs or semptoms plus radiological imaging/NA	SO-to-FMC time, Laboratory values, Primary angioplasty, Thrombus aspiration, Gp2b3a inhibitor use, Baseline thrombus grade, Modified thrombus grade, TIMI status, In-hospital mortality, Bleeding, Stent thrombosis, Cardiogenic shock
			Non-COVID-19 STEMI	*n* = 668, age 60.0 ± 12.3 years, 78% males	11/3/2020–15/5/2020		
Koutsoukis et al. (21)	France	Multicentric cross-sectional study	COVID-19 STEMI	*n* = 17, age 63.4 ± 13.2 years, 70% males	1/4/2020–22/4/2020	RT-PCR on nasopharyngeal samples/NA	Laboratory values, Primary angioplasty, Thrombus aspiration, MINOCA, Stent implantation, Gp2b3a inhibitor use, In-hospital mortality
			Non-COVID-19 STEMI	*n* = 99, age 63.8 ± 13.9 years, 67% males	1/4/2020–22/4/2020		
Garcia et al. (22)	USA & Canada	Multicentric cohort study	COVID-19 STEMI	*n* = 230, 71% males	1/1/2020–6/12/2020	Comfirmed COVID+ by any commercially available test/NA	D2B time, Primary angioplasty, MINOCA, In-hospital mortality, LOS
			Non-COVID-19 STEMI	*n* = 460, 68% males	1/2015–12/2019		
Kite et al. (23)	Data from 55 international centers	Multicentric corhort study	COVID-19 STEMI	*n* = 144, age 63.1 ± 12.6 years, 77.8% males	1/3/2020–31/7/2020	RT-PCR or clinical status plus CXR or CT findings/NA	D2B time, Laboratory values, Thrombus aspiration, In-hospital mortality, Bleeding, Cardiogenic shock, LOS
			Non-COVID-19 STEMI	*n* = 24961, age 65.6 ± 13.4 years, 72.2% males	2018–2019		
Little et al. (24)	UK	Multicentric cohort study	COVID-19 STEMI	*n* = 46, age 61.80 ± 7.95 years, 80.4% males	1/3/2020–30/4/2020	RT-PCR on oro/nasopharyngeal throat swabs or typical symptoms plus radiographic appearances and characteristic blood test/NA	D2B time, Laboratory values, Thrombus aspiration, Gp2b3a inhibitor use, TIMI status, In-hospital mortality, Cardiogenic shock, ICU admission, LOS
			Non-COVID-19 STEMI	*n* = 302, age 64.18 ± 13.41 years, 79.8% males	1/3/2020–30/4/2020		
Marfella et al. (25)	Italy	Multicentric cohort study	COVID-19 STEMI	*n* = 46, age 56.13 ± 6.21 years, 67.4% males	2/2020–11/2020	RT-PCR on nasal/pharyngeal swabs/NA	D2B time, Laboratory values, Gp2b3a inhibitor use, Modified thrombus grade, TIMI status, In-hospital mortality, LOS, ICU admission, Cardiogenic shock
			Non-COVID-19 STEMI	*n* = 130, age 68.43 ± 6.46 years, 66.2% males	2/2020–11/2020		
Pellegrini et al. (26)	Italy	Monocentric cohort study	COVID-19 STEMI	*n* = 24, age 69.63 ± 11.00 years, 83.3% males	8/3/2020–20/4/2020	RT-PCR on nasal swab or endotracheal aspirate/3–6 h	Thrombus aspiration, MINOCA, Stent implantation, Gp2b3a inhibitor use, In-hospital mortality, Cardiogenic shock, Bleeding
			Non-COVID-19 STEMI	*n* = 26, age 64.65 ± 13.04 years, 84.6% males	8/3/2020–20/4/2020		
Rodriguez-Leor et al. (27)	Spain	Multicentric cohort study	COVID-19 STEMI	*n* = 91, age 64.8 ± 11.8 years, 84.4% males	14/3/2020–30/4/2020	PCR assay/NA	SO-to-FMC time, Primary angioplasty, Thrombus aspiration, MINOCA, Stent implantation, Gp2b3a inhibitor use, TIMI status, In-hospital mortality, Cardiogenic shock, Stent thrombosis, bleeding
			Non-COVID-19 STEMI	*n* = 919, age 62.5 ± 13.1 years, 78.4% males	14/3/2020–30/4/2020		
Choudry et al. (28)	UK	Monocentric cohort study	COVID-19 STEMI	*n* = 39, age 61.7 ± 11.0 years, 84.6% males	1/3/2020–20/5/2020	PT-PCR on nasal/ pharyngeal swabs/NA	D2B time, Laboratory values, Primary angioplasty, Thrombus aspiration, Gp2b3a inhibitor use, Baseline thrombus grade, Modified thrombus grade, TIMI status, In-hospital mortality, Stent thrombosis
			Non-COVID-19 STEMI	*n* = 76, age 61.7 ± 12.6 years, 75% males	1/3/2020–20/5/2020		
Blasco et al. (29)	Spain	Monocentric cross-sectional study	COVID-19 STEMI	*n* = 5, age 62 ± 14 years, 80% males	23/3/2020–11/4/2020	RT-PCR on nasopharyngeal and throat swab samples/NA	Laboratory values
			Non-COVID-19 STEMI	*n* = 50, age 58 ± 12 years, 88% males	7/2015–12/2015		
Güler et al. (30)	Turkey	Monocentric cross-sectional study	COVID-19 STEMI	*n* = 62, age 60.2 ± 9.5 years, 66.1% males	11/3/2020–10/1/2021	RT-PCR on nasopharyngeal swabs / NA	SO-to-FMC time, D2B time, Laboratory values, Thrombus aspiration, Gp2b3a inhibitor use, Baseline thrombus grade, TIMI status, In-hospital mortality, ICU admission, LOS
			Non-COVID-19 STEMI	*n* = 64, age 63 ± 8 years, 70.3% males	11/3/2020–10/1/2021		

*UK, United Kingdom; NOS, Newcastle-Ottawa Scale; D2B, door to balloon; MINOCA, myocardial infarction with non-obstructive coronary arteries; TIMI, thrombolysis in myocardial infarction; SO-to-FMC, symptom onset to first medical contact; LOS, length of stay; ICU, intensive care unit; RT-PCR, reverse transcriptase-polymerase chain reaction; CT, computed tomography; CXR, chest x-ray*.

**Table 2B:** Baseline characteristics of study subjects.

**References**	**Study group**	**Total subjects (n)**	**Age (years) (mean ±SD)**	**Male (%)**	**Body mass index (kg/m^**2**^)**	**Diabetes mellitus (%)**	**Hypertension (%)**	**Dyslipidemia (%)**	**Smoking (%)**	**Multivessel desease (%)**	**Previous myocardial infarction (%)**
Popovic et al. (18)	COVID-19 STEMI	11	63.6 ± 17.4	63.9	25.1 ± 8.1	18.2	45.5	27.3	36.4	0	NA
	Non-COVID-19 STEMI	72	62.5 ± 12.6	73.6	27.02 ± 4.8	19.4	43.1	38.9	55.6	12.5	NA
Siudak et al. (19)	COVID-19 STEMI	145	63.19 ± 12.55	71.33	NA	14.48	46.21	NA	37.24	NA	12.41
	Non-COVID-19 STEMI	2,276	65.43 ± 12.23	67.65	NA	16.86	57.55	NA	31.08	NA	15.94
Kiris et al. (20)	COVID-19 STEMI	65	66.8 ± 12.0	68	NA	26	48	NA	34	44	NA
	Non-COVID-19 STEMI	668	60.0 ± 12.3	78	NA	29	42	NA	33	40	NA
Koutsoukis et al. (21)	COVID-19 STEMI	17	63.4 ± 13.2	70	NA	NA	NA	NA	NA	30.7	NA
	Non-COVID-19 STEMI	99	63.8 ± 13.9	67	NA	NA	NA	NA	NA	61.2	NA
Garcia et al. (22)	COVID-19 STEMI	230	18–55 yrs: 23%; 55–65 yrs: 32%; 66–75 yrs: 28%; >75 yrs: 17%	71	29.3 ± 7.6	46	73	46	44	0	13
	Non-COVID-19 STEMI	460	18–55 yrs: 26%; 55–65 yrs: 30%; 66–75 yrs: 27%; >75 yrs: 17%	68	29.5 ± 6.4	28	69	60	59	16	24
Kite et al. (23)	COVID-19 STEMI	144	63.1 ± 12.6	77.8	27.3 ± 4.5	34	64.8	46	31.7	NA	16.4
	Non-COVID-19 STEMI	24,961	65.6 ± 13.4	72.2	27.8 ± 5.5	20.9	44.8	28.9	33.7	NA	13
Little et al. (24)	COVID-19 STEMI	46	61.80 ± 7.95	80.4	NA	32.6	54	52.2	41.3	NA	10.9
	Non-COVID-19 STEMI	302	64.18 ± 13.41	79.8	NA	23.5	50.7	33.1	41.7	NA	12.6
Marfella et al. (25)	COVID-19 STEMI	46	56.13 ± 6.21	67.4	27.09 ± 1.81	17.4	39.1	15.2	6.5	NA	NA
	Non-COVID-19 STEMI	130	68.43 ± 6.46	66.2	29.55 ± 1.97	29.2	55.4	23.7	29.2	NA	NA
Pellegrini et al. (26)	COVID-19 STEMI	24	69.63 ± 11.00	83.3	26.60 ± 3.36	41.7	70.8	62.5	29.2	45.8	29.2
	Non-COVID-19 STEMI	26	64.65 ± 13.04	84.6	26.11 ± 3.43	15.4	53.9	65.4	38.5	28.6	19.2
Rodriguez-Leor et al. (27)	COVID-19 STEMI	91	64.8 ± 11.8	84.4	NA	23.1	51.7	48.4	18.7	37.4	NA
	Non-COVID-19 STEMI	919	62.5 ± 13.1	78.4	NA	20.9	53.3	46.9	45.5	37.1	NA
Choudry et al. (28)	COVID-19 STEMI	39	61.7 ± 11.0	84.6	26.7 (24.8–30.7)	46.2	71.8	61.6	61.6	NA	15.4
	Non-COVID-19 STEMI	76	61.7 ± 12.6	75	26.7 (24.8–30.7)	46.2	42.1	36.8	46.1	NA	3.9
Blasco et al. (29)	COVID-19 STEMI	5	62 ± 14	80	28.0 (27.3–30.1)	0	80	0	40	NA	NA
	Non-COVID-19 STEMI	50	58 ± 12	88	27.6 (24.9–30.3)	8	42	52	78	NA	NA
Güler et al. (30)	COVID-19 STEMI	62	60.2 ± 9.5	66.1	NA	48.4	59.7	43.5	51.6	NA	9.7
	Non-COVID-19 STEMI	64	63 ± 8	70.3	NA	54.7	57.8	34.3	56.3	NA	28.1

### Delays

**Figure 2:**
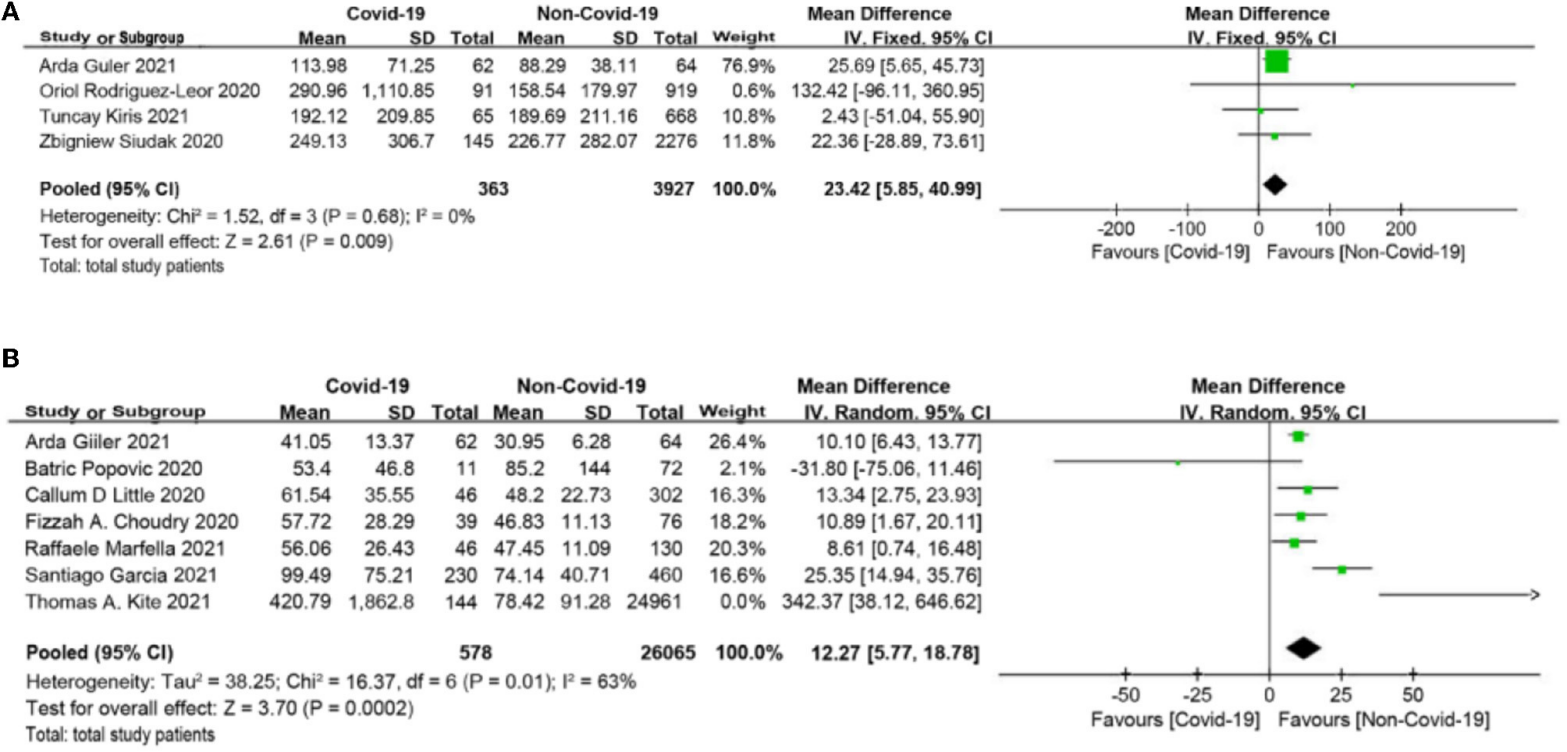
**(A)** Symptom onset to first medical contact (SO-to-FMC) time forest plot (minutes). **(B)** Door to balloon (D2B) time forest plot (minutes).

The symptom onset to first medical contact (SO-to-FMC) time among STEMI, which was reported in four studies (19, 20, 27, 30), was significantly different between the COVID-19 group and the non-COVID-19 group (MD = 23.42 min, 95% CI: 5.85 to 40.99 min, *p* = 0.009; Figure 2A). Furthermore, seven studies (18, 22–25, 28, 30) reported the time from door to balloon (D2B) and found that D2B was significantly longer in the COVID-19 group (MD = 12.27 min, 95% CI: 5.77 to 18.78 min, *p* = 0.0002; Figure 2B) than in the non-COVID-19 group. *3.3 Laboratory values*.

**Figure 3:**
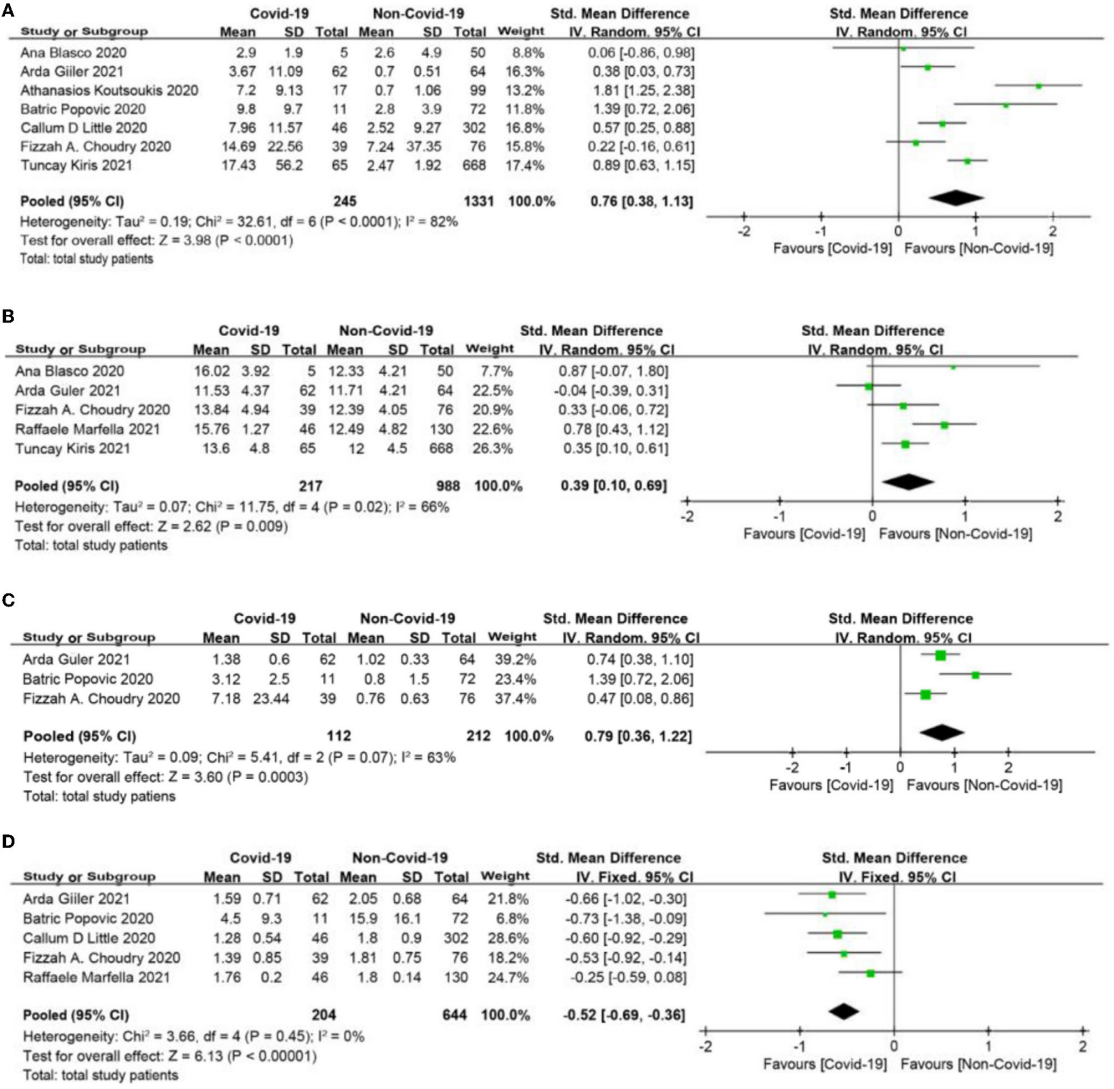
**(A)** C-reactive protein (CRP) forest plot (mg/dl). **(B)** White blood cell **(**WBC) forest plot (*10^9^/L). **(C)** D-dimer forest plot (mg/L). **(D)** Lymphocyte count forest plot (*10^9^/L).

The meta-analysis showed that compared to the non-COVID-19 group, the COVID-19 group had significantly higher levels of C-reactive protein (CRP), white blood cell count (WBC), and D-dimer (SMD = 0.76, 95% CI: 0.38 to 1.13, *p* < 0.0001; SMD = 0.39, 95% CI: 0.1 to 0.69, *p* = 0.009; SMD = 0.79, 95% CI: 0.36 to 1.22, *p* = 0.0003, respectively, Figures 3A–C), and had significantly lower level of lymphocyte count (SMD = −0.52, 95% CI: −0.69, −0.36, *p* < 0.0001, Figure 3D).

### Management and Procedural Characteristic

**Figure 4:**
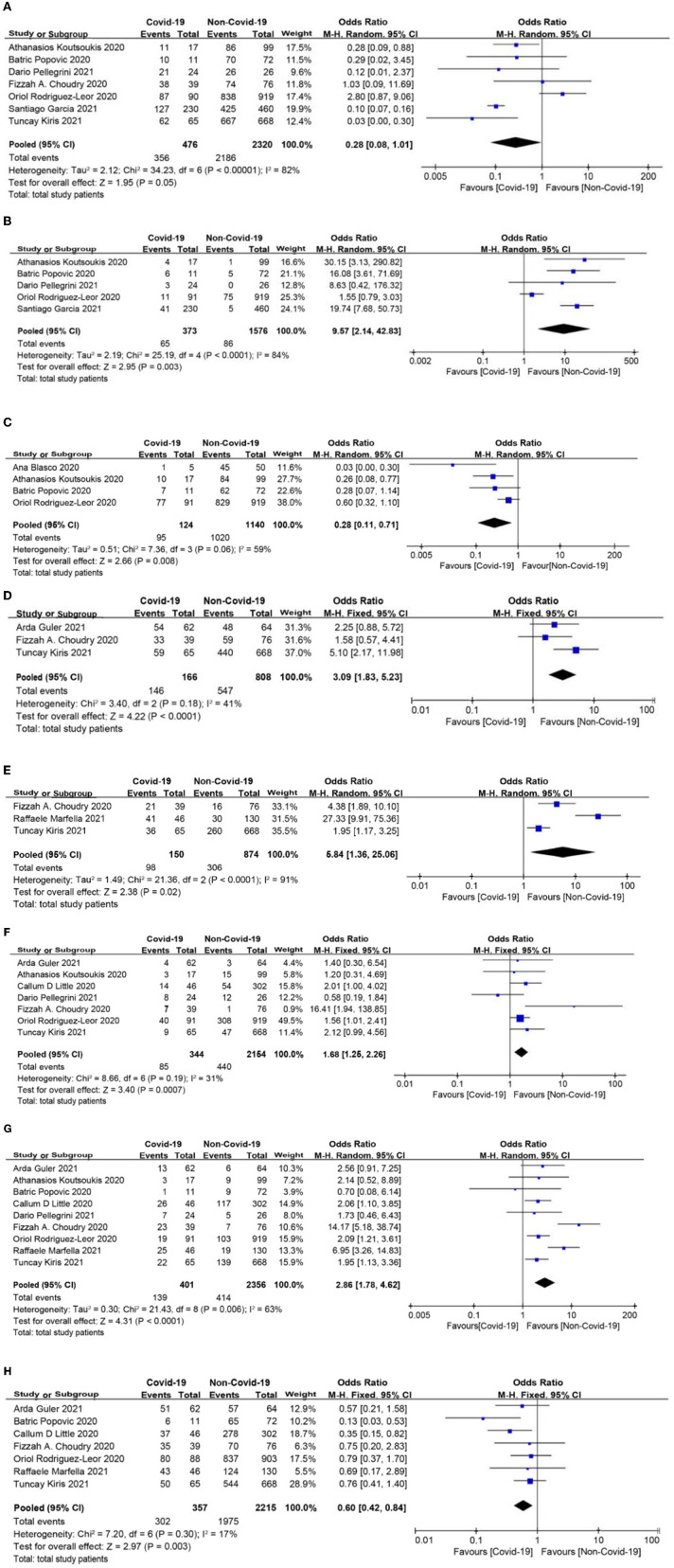
**(A)** Primary angioplasty forest plot. **(B)** Myocardial infarction with no obstructive coronary atherosclerosis (MINOCA) forest plot. **(C)** Stent implantation forest plot. **(D)** Baseline thrombus grade forest plot. **(E)** Modified thrombus grade forest plot. **(F)** Thrombus aspiration forest plot. **(G)** Glycoprotein IIbIIIa (Gp2b3a) inhibitor use forest plot. **(H)** Thrombolysis in myocardial infarction (TIMI)-3 flow forest plot.

There was no significant difference in the rate of primary angioplasty between the two groups (OR = 0.28, 95% CI: 0.08 to 1.01, *p* = 0.05; Figure 4A). Myocardial infarction with no obstructive coronary atherosclerosis (MINOCA) was more frequently observed, and the rate of stent implantation was lower in patients with COVID-19 infection (OR = 9.57, 95% CI: 2.14 to 42.83, *p* = 0.003; OR = 0.28, 95% CI: 0.11 to 0.71, *p* = 0.008, respectively, Figures 4B,C). Baseline thrombus grade > 3 and modified thrombus grade > 3 were significantly higher in the COVID-19 group than in the non-COVID-19 group (OR = 3.09, 95% CI: 1.83 to 5.23, *p* < 0.0001; OR = 5.84, 95% CI: 1.36 to 25.06, *p* = 0.02, respectively; Figures 4D,E). Intracoronary thrombus was angiographically identified and scored in 0–5 grades as previously described (31). In patients initially presenting with grade 5, thrombus grade will be reclassified into one of the other categories after flow achievement (32). After reclassification and based on clinical outcomes, the thrombus burden can be divided into 2 categories: low thrombus grade for thrombus < grade 4, and high thrombus grade for thrombus grade 4 (32). Consistent with this, the COVID-19 group showed a higher use of thrombus aspiration and glycoprotein IIbIIIa (Gp2b3a) inhibitor (OR = 1.68, 95% CI: 1.25 to 2.26, *p* = 0.0007; OR = 2.86, 95% CI: 1.78 to 4.62, *p* < 0.0001, respectively; Figures 4F,G). Moreover, thrombolysis in myocardial infarction (TIMI)-3 flow post-procedure was less common in the COVID-19 group than in the non-COVID-19 group (OR = 0.6, 95% CI: 0.42 to 0.84, *p* = 0.003, Figure 4H).

### In-Hospital Outcomes

**Figure 5:**
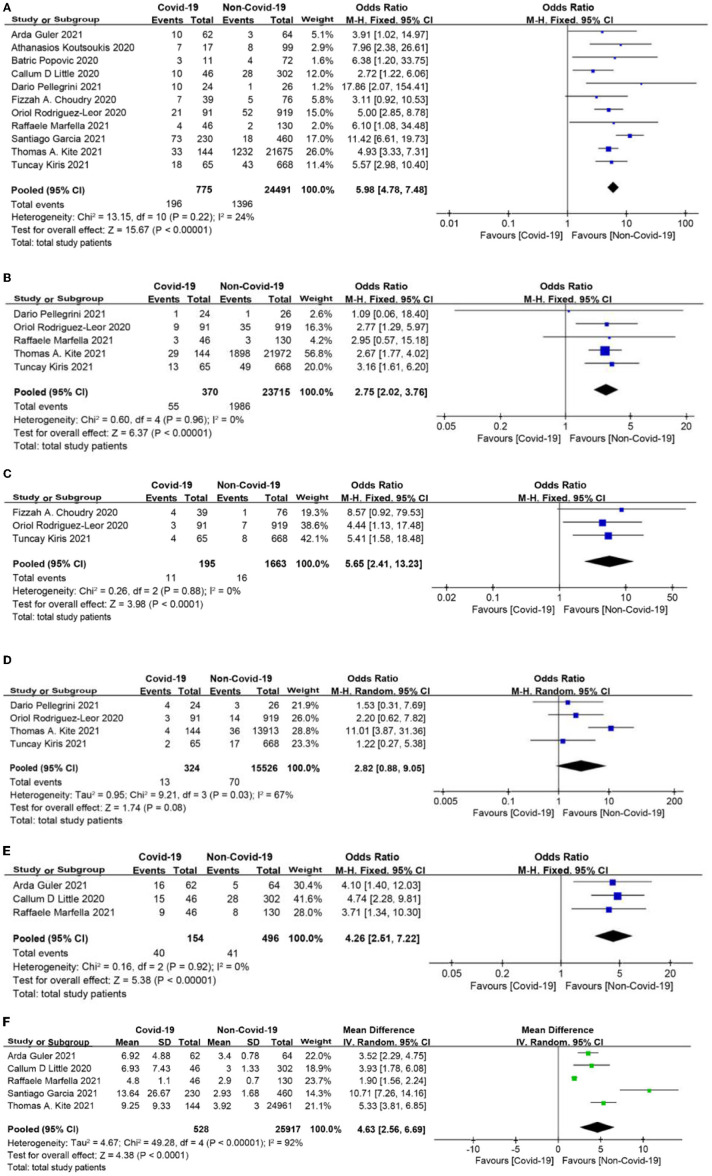
**(A)** In-hospital mortality forest plot. **(B)** Cardiogenic shock forest plot. **(C)** Stent thrombosis forest plot. **(D)** Bleeding forest plot. **(E)** Intensive care unit (ICU) admission rate forest plot. **(F)** Length of stay forest plot (days).

In-hospital mortality among patients with COVID-19 was significantly higher than that in patients without COVID-19 (OR = 5.98, 95% CI: 4.78 to 7.48, *p* < 0.0001, Figure 5A). The rates of cardiogenic shock as well as stent thrombosis were also higher in the COVID-19 group than in the non-COVID-19 group (OR = 2.75, 95% CI: 2.02 to 3.76, *p* < 0.0001; OR = 5.65, 95% CI: 2.41 to 13.23, *p* < 0.0001, respectively; Figures 5B,C). Although bleeding was more common in STEMI patients with COVID-19, there was no significant difference between the two groups (OR = 2.82, 95% CI: 0.88 to 9.05, *p* = 0.08, Figure 5D). In addition, patients with COVID-19 were more likely to be admitted to the intensive care unit (ICU) and had a longer length of hospital stay (OR = 4.26, 95% CI: 2.51 to 7.22, *p* < 0.0001; MD = 4.63 days, 95% CI: 2.56 to 6.69 days, *p* < 0.0001, respectively, Figures 5E,F).

### Grade Summary of Findings

The GRADE summary of findings tool was used to evaluate the quality of evidence, and the assessment for each outcome is presented in Table 3. In addition to in-hospital mortality, which moderates the quality of evidence, other outcomes had low or very low quality of evidence because all included studies were observational.

**Table 3:** GRADE summary of findings.

**Effects of COVID-19 in STEMI patients**						
**Patient or population:** STEMI Patients **Setting:** Europe, Asian, North America **Intervention:** COVID-19 **Comparison:** Non-COVID-19						
**Outcomes**	Anticipated absolute effects*(95% CI)		**Relative effect (95% CI)**	**No of participants (studies)**	**Certainty of the evidence (GRADE)**	**Comments**
	**Risk with Non-COVID-19**	**Risk with COVID-19**				
Symptom-to-FMC time	The mean symptom-to-FMC time was 0	MD 23.42 higher (5.85 higher to 40.99 higher)	–	4,290 (4 observational studies)	⊕○○○ Very low	NA
D2B time	The mean D2B time was 0	MD 12.27 higher (5.77 higher to 18.78 higher)	–	26,643 (7 observational studies)	⊕○○○ Very low	NA
CRP	–	SMD 0.76 higher (0.38 higher to 1.13 higher)	–	1,576 (7 observational studies)	⊕○○○ Very low	NA
WBC	–	SMD 0.39 higher (0.1 higher to 0.69 higher)	–	1,205 (5 observational studies)	⊕○○○ Very low	NA
D–Dimer	–	SMD 0.79 higher (0.36 higher to 1.22 higher)	–	324 (3 observational studies)	⊕○○○ Very low	NA
Lymphocyte count	–	SMD 0.52 lower (0.69 lower to 0.36 lower)	–	848 (5 observational studies)	⊕⊕○○ Low	NA
Primary angioplasty	942 per 1,000	820 per 1,000 (566 to 943)	OR 0.28 (0.08 to 1.01)	2,796 (7 observational studies)	⊕○○○ Very low	NA
MINOCA	55 per 1,000	356 per 1,000 (110 to 712)	OR 9.57 (2.14 to 42.83)	1,949 (5 observational studies)	⊕○○○ Very low	NA
Stent implantation	895 per 1,000	704 per 1,000 (483 to 858)	OR 0.28 (0.11 to 0.71)	1,264 (4 observational studies)	⊕○○○ Very low	NA
Baseline thrombus grade > 3	677 per 1,000	866 per 1,000 (793 to 916)	OR 3.09 (1.83 to 5.23)	974 (3 observational studies)	⊕○○○ Very low	NA
Modified thrombus grade > 3	350 per 1,000	759 per 1,000 (423 to 931)	OR 5.84 (1.36 to 25.06)	1,024 (3 observational studies)	⊕○○○ Very low	NA
Thrombus aspiration	204 per 1,000	301 per 1,000 (243 to 367)	OR 1.68 (1.25 to 2.26)	2,498 (7 observational studies)	⊕⊕○○ Low	NA
Gp2b3a inhibitor	176 per 1,000	379 per 1,000 (275 to 496)	OR 2.86 (1.78 to 4.62)	2,757 (9 observational studies)	⊕○○○ Very low	NA
TIMI-3 Flow	892 per 1,000	832 per 1,000 (776 to 874)	OR 0.60 (0.42 to 0.84)	2,572 (7 observational studies)	⊕⊕○○ Low	NA
In- hospital mortality	57 per 1,000	265 per 1,000 (224 to 311)	OR 5.98 (4.78 to 7.48)	25,266 (11 observational studies)	⊕⊕⊕○ Moderate	NA
Cardiogenic shock	84 per 1,000	201 per 1,000 (156 to 256)	OR 2.75 (2.02 to 3.76)	24,085 (5 observational studies)	⊕⊕○○ Low	NA
Stent thrombosis	10 per 1,000	52 per 1,000 (23 to 114)	OR 5.65 (2.41 to 13.23)	1,858 (3 observational studies)	⊕⊕○○ Low	NA
Bleeding	5 per 1,000	13 per 1,000 (4 to 39)	OR 2.82 (0.88 to 9.05)	15,850 (4 observational studies)	⊕○○○ Very low	NA
ICU admission	83 per 1,000	277 per 1,000 (184 to 394)	OR 4.26 (2.51 to 7.22)	650 (3 observational studies)	⊕○○○ Very low	NA
Length of stay	The mean length of stay was 0	MD 4.63 higher (2.56 higher to 6.69 higher)	-	26,445 (5 observational studies)	⊕○○○ Very low	NA

### Sensitivity Analysis and Publication Bias

**Figure 6:**
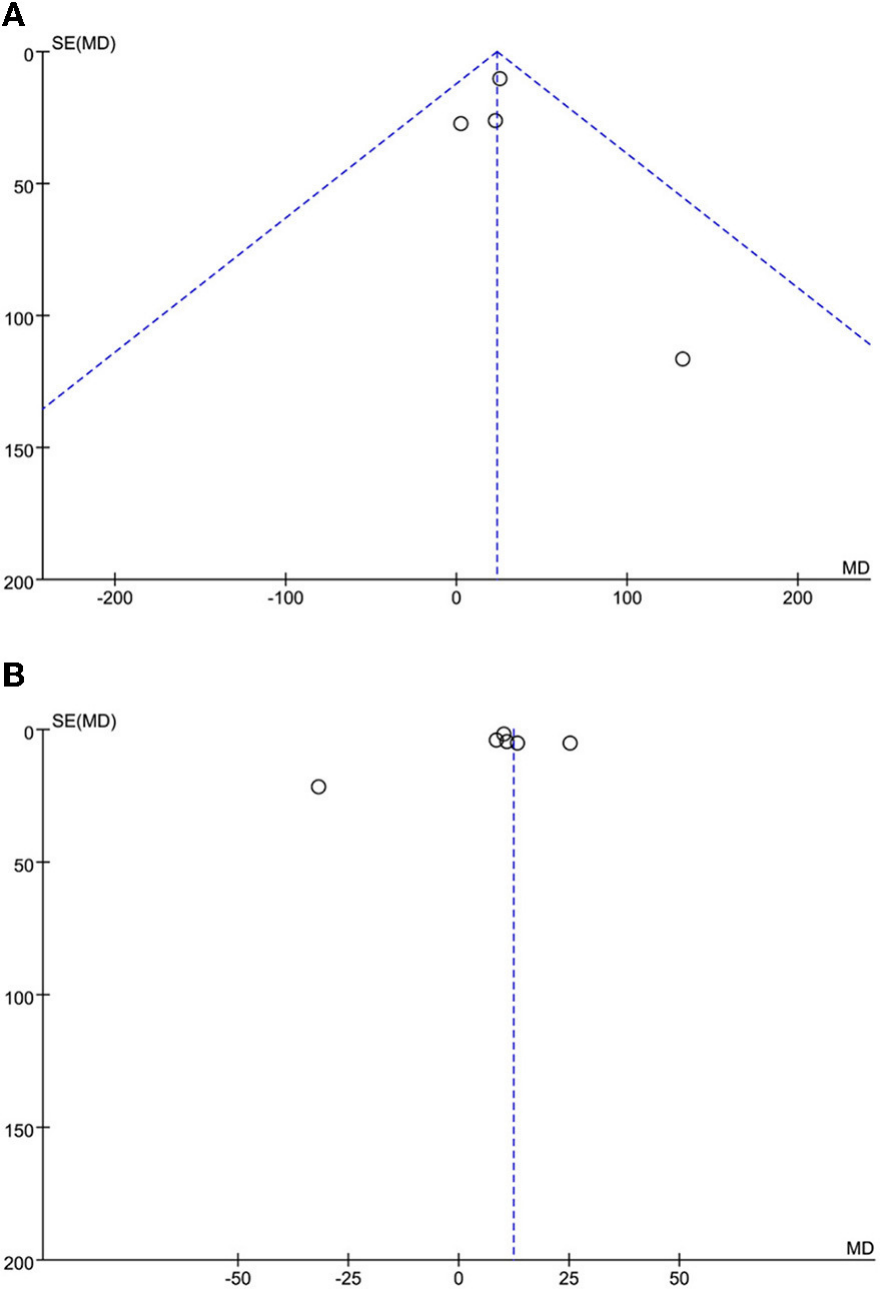
**(A)** SO-to-FMC time funnel plot. **(B)** D2B time funnel plot.

**Figure 7:**
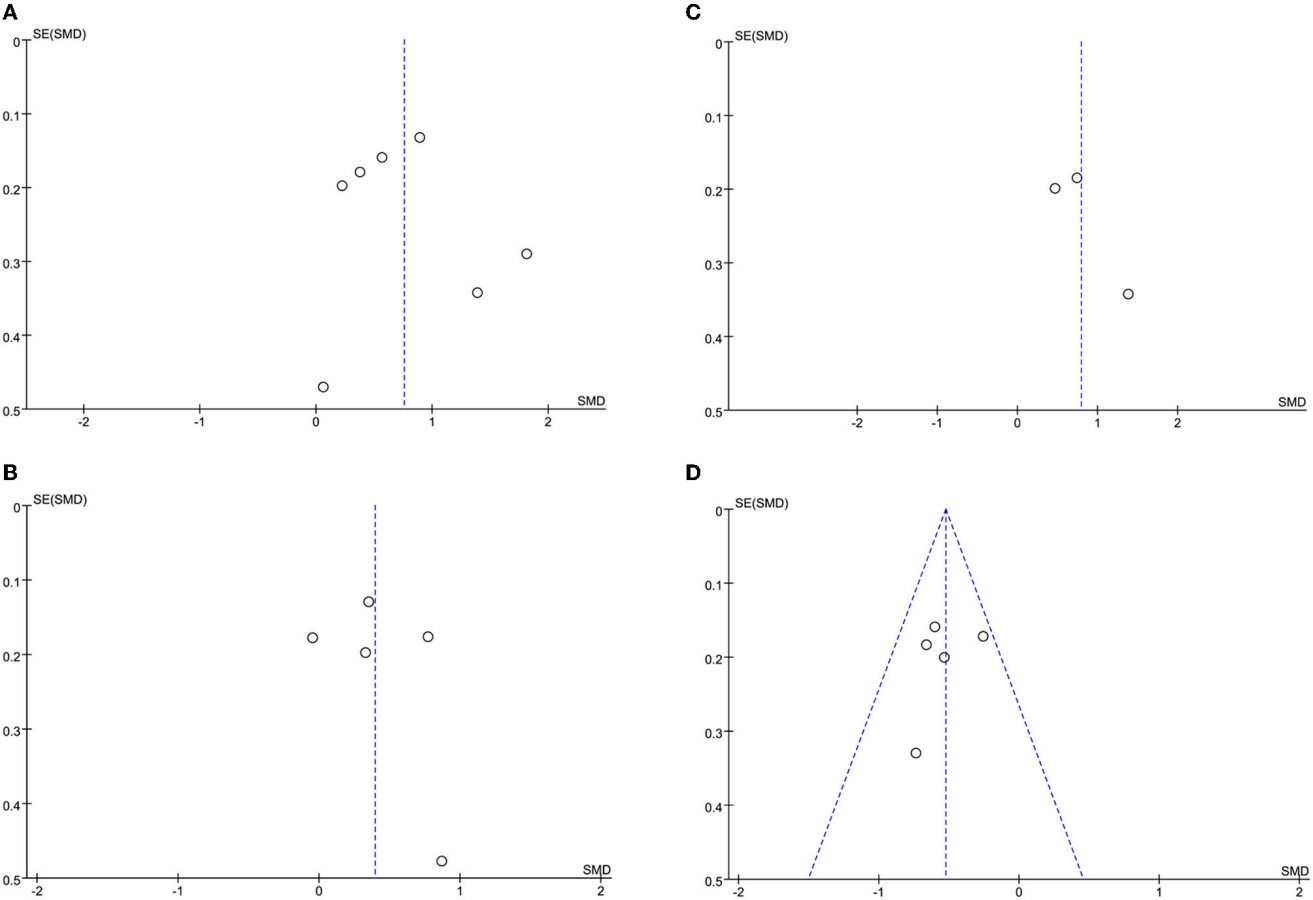
**(A)** CRP funnel plot**. (B)** WBC funnel plot. **(C)** D-dimer funnel plot. **(D)** Lymphocyte count funnel plot.

**Figure 8:**
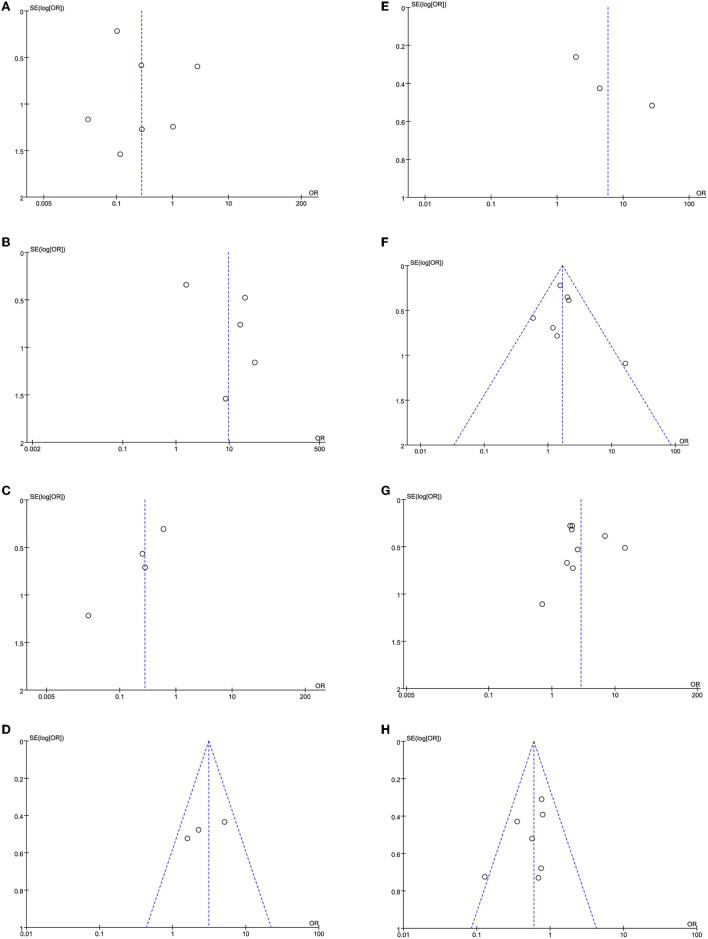
**(A)** Primary angioplasty funnel plot. **(B)** MINOCA funnel plot. **(C)** Stent implantation funnel plot. **(D)** Baseline thrombus grade funnel plot. **(E)** Modified thrombus grade funnel plot. **(F)** Thrombus aspiration funnel plot. **(G)** Gp2b3a inhibitor use funnel plot. **(H)** TIMI-3 flow funnel plot.

**Figure 9:**
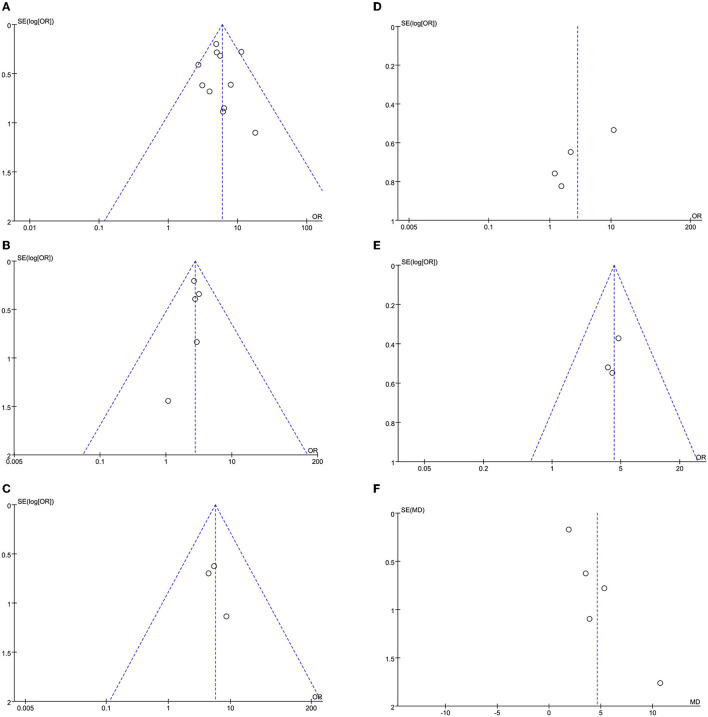
**(A)** In-hospital mortality funnel plot. **(B)** Cardiogenic shock funnel plot. **(C)** Stent thrombosis funnel plot. **(D)** Bleeding funnel plot. **(E)** ICU admission rate funnel plot. **(F)** Length of stay funnel plot.

The leave-one-out approach was applied for sensitivity analysis to evaluate the impact of a single study on outcomes with a high degree of heterogeneity. As shown in Table 4, the overall results were relatively robust and not influenced by a single study, except for primary angioplasty, stent implantation, and modified thrombus grade. An asymmetrical plot was observed in some funnel plots, suggesting that publication bias may exist (Figures 6A–9F).

**Table 4:** Leave-one-out analysis.

**Study name**	**Statistics with study excluded**		
	**Odds ratio or SMD**	**95% CI**	***P*-value**
**D2B time**			
Güler et al. (30)	12.66	2.96 to 22.35	0.01
Popovic et al. (18)	13.06	7.13 to 18.99	<0.0001
Little et al. (24)	12.01	4.16 to 19.86	0.003
Choudry et al. (28)	12.52	4.35 to 20.68	0.003
Marfella et al. (25)	13.1	4.66 to 21.54	0.002
Garcia et al. (22)	9.92	4.47 to 15.35	0.0004
Kite et al. (23)	12.15	6.47 to 17.82	<0.0001
**CRP**			
Blasco et al. (29)	0.82	0.43 to 1.21	<0.0001
Güler et al. (30)	0.83	0.40 to 1.26	0.0002
Koutsoukis et al. (21)	0.59	0.29 to 0.90	0.0001
Popovic et al. (18)	0.67	0.28 to 1.06	0.0007
Little et al. (24)	0.8	0.33 to 1.26	0.0007
Choudry et al. (28)	0.86	0.45 to 1.26	<0.0001
Kiris et al. (20)	0.73	0.27 to 1.20	0.002
**WBC**			
Blasco et al. (29)	0.35	0.04 to 0.67	0.03
Güler et al. (30)	0.5	0.25 to 0.76	<0.0001
Choudry et al. (28)	0.42	0.04 to 0.81	0.03
Marfella et al. (25)	0.26	0.08 to 0.44	0.004
Kiris et al. (20)	0.038	0.18 to 0.59	0.0002
**D-Dimer**			
Güler et al. (30)	0.89	0.01 to 1.78	0.05
Popovic et al. (18)	0.62	0.35 to 0.88	<0.0001
Choudry et al. (28)	1.00	0.38 to 1.62	0.002
**Primary Angioplasty**			
Koutsoukis et al. (21)	0.27	0.05 to 1.43	0.12
Popovic et al. (18)	0.28	0.07 to 1.15	0.08
Pellegrini et al. (26)	0.31	0.01 to 1.24	0.10
Choudry et al. (28)	0.23	0.06 to 0.94	0.04
Rodriguez-Leor et al. (27)	0.12	0.08 to 0.17	<0.0001
Garcia et al. (22)	0.36	0.09 to 1.49	0.16
Kiris et al. (20)	0.21	0.16 to 0.29	<0.0001
**MINOCA**			
Koutsoukis et al. (21)	7.63	1.44 to 40.43	0.02
Popovic et al. (18)	8.49	1.37 to 52.74	0.02
Pellegrini et al. (26)	9.81	1.84 to 52.38	0.01
Rodriguez-Leor (27)	18.62	8.73 to 39.72	<0.0001
Garcia et al. (22)	7.56	1.38 to 41.37	0.02
**Stent Implantation**			
Blasco et al. (29)	0.46	0.28 to 0.75	0.002
Koutsoukis et al. (21)	0.25	0.06 to 1.01	0.05
Popovic et al. (18)	0.25	0.07 to 0.90	0.03
Rodriguez-Leor et al. (27)	0.20	0.09 to 0.43	<0.0001
**Modified Thrombus Grade**			
Choudry et al. (28)	7.03	0.52 to 96.03	0.14
Marfella et al. (25)	2.72	1.25 to 5.94	0.01
Kiris et al. (20)	10.69	1.75 to 65.11	0.01
**Gp2b3a inhibitor use**			
Güler et al. (30)	2.90	1.70 to 4.93	<0.0001
Koutsoukis et al. (21)	2.93	1.75 to 4.90	<0.0001
Popovic et al. (18)	3.03	1.87 to 4.93	<0.0001
Little et al. (24)	3.02	1.72 to 5.30	0.0001
Pellegrini et al. (26)	2.99	1.79 to 5.01	<0.0001
Choudry et al. (28)	2.37	1.81 to 3.11	<0.0001
Rodriguez-Leor et al. (27)	2.93	2.19 to 3.92	<0.0001
Marfella et al. (25)	2.41	1.83 to 3.17	<0.0001
Kiris et al. (20)	3.01	2.25 to 4.03	<0.0001
**Bleeding**			
Pellegrini et al. (26)	3.30	0.77 to 14.07	0.11
Rodriguez-Leor et al. (27)	2.95	0.55 to 15.73	0.21
Kite et al. (23)	1.62	0.71 to 3.73	0.25
Kiris et al. (20)	3.62	0.92 to 14.23	0.07
**Length of Stay**			
Güler et al. (30)	5.11	2.17 to 8.06	0.0007
Little et al. (24)	4.84	2.41 to 7.27	<0.0001
Marfella et al. (25)	5.42	3.24 to 7.26	<0.0001
Garcia et al. (22)	3.56	1.85 to 5.27	<0.0001
Kite et al. (23)	4.41	2.14 to 6.69	0.0001

## Discussion

### Clinical Implications

This is the first meta-analysis to compare the characteristics, management, and clinical outcomes of patients with STEMI presenting with COVID-19 infection and that of those patients without COVID-19 infection. Compared to the non-COVID-19 group, the COVID-19 group had significant delays in SO-to-FMC and D2B times. Among the two groups, laboratory values, such as CRP, WBC, and D-dimer, were elevated in the COVID-19 group, while lymphocyte count was found to be lower compared to the non-COVID-19 group. In addition, STEMI concomitant with COVID-19 infection was characterized by a higher rate of MINOCA, lower rate of stent implantation, and higher thrombus grade, and associated higher use of thrombus aspiration and Gp2b3a inhibitors. Furthermore, we found that the COVID-19 group had an increased rate of in-hospital mortality, cardiogenic shock, stent thrombosis, ICU admission, longer length of hospital stays, and decreased TIMI flow post-procedure.

The COVID-19 pandemic started in late 2019 and has caused severe delays in the treatment of patients with STEMI compared to the pre-COVID-19 era, and this is mostly explained by the limited access to emergency medical services (EMS) and the lack of effective organization of healthcare systems (33, 34). Several studies reported that the time from SO-to-FMC and D2B was longer in STEMI patients with COVID-19 than in those without COVID-19, which may be related to the following factors: a higher rate of respiratory symptoms without chest pain as a clinical manifestation in COVID-19 patients may result in an unclear diagnosis of heart attack and lead to a delay in seeking medical service (35), Furthermore, interventional procedures may be more complex in COVID-19 patients than in non-COVID-19 patients (24).

The reperfusion strategy for patients with STEMI during the COVID-19 pandemic remains controversial. The Chinese Cardiac Society and the Canadian Association of Interventional Cardiology recommend thrombolysis as the preferred reperfusion strategy for patients with STEMI (36, 37). In contrast, the American College of Cardiology (ACC) and the Society for Cardiovascular Angiography and Interventions (SCAI) still suggested the use of primary percutaneous coronary intervention (PPCI) as the main treatment for all patients with STEMI during the COVID-19 crisis (1, 2). Rashid et al. reported that STEMI patients with COVID-19 were less likely to receive PPCI than STEMI patients without COVID-19 (38). However, in this study, we did not find a significant difference in the rate of primary angioplasty between both groups. Moreover, we found that the COVID-19 group had a lower rate of stent implantation, which may be associated with a higher rate of MINOCA.

Previous studies have shown that COVID-19 may lead to a prothrombotic state and that a high thrombus burden is more common in STEMI patients with COVID-19 (39–42). SARS-CoV-2 causes a systemic inflammatory response, resulting in endothelial and hemostatic activation, which involves the activation of platelets and the coagulation cascade (43). In addition, our study found that the time from SO-to-FMC and D2B was longer in STEMI patients with COVID-19 than in those without COVID-19. The studies of Duman et al. (44) and Ge et al. (45) reported that the delay in SO-to-FMC and D2B would prolong the time for opening infarct-related vessels which may account for a higher thrombus burden. Therefore, in the COVID era, it is of great significance that novel technologies should be developed so as to achieve more efficient thrombus aspiration in patients with very high intra-coronary thrombus burden such as patients with STEMI and coexistent COVID-19 infection (46). Furthermore, strategies to reduce reperfusion delay times such as educating the public about the recognition and diversity of coronary symptoms and optimizing interventional procedures are essential. In keeping with the high thrombus burden, the COVID-19 group had elevated CRP, WBC, and D-dimer levels and a lower lymphocyte count compared to the non-COVID-19 group. High thrombus grade, reduced TIMI flow, high rate of MINOCA, and stent thrombosis may be the result of the intense inflammatory and heightened thrombus burden observed in COVID-19 patients (18, 27, 28, 34). Consistently, the data presented here demonstrated a more aggressive use of thrombus aspiration and a Gp2b3a inhibitor in STEMI patients with concomitant SARS-CoV-2 infection. The use of a Gp2b3a inhibitor may also increase the risk of bleeding (47), but this study showed no significant difference between the two groups in terms of bleeding.

Hospital-mortality was dramatically higher in STEMI patients who presented with COVID-19 than in those without COVID-19. Longer ischemia time, higher thrombus burden, and increased rate of adverse cardiovascular events, including cardiogenic shock, may also be contributory (48, 49). Current studies (50, 51) have reported that STEMI patients with concomitant COVID-19 have higher ICU admission rates and longer lengths of stay, and the results of this meta-analysis support this finding. An increased ICU admission rate and length of stay may have a significant impact on hospital resources. Taken together, COVID-19 status may have great implications on the characteristics, management, and outcomes of patients with STEMI.

### Heterogeneity of Meta-Analysis

In a meta-analysis, heterogeneity may exist while the sample estimates for the population risk were of different magnitudes (52). The *I*^2^ statistic means the percentage of total variation across effect size estimates that is due to heterogeneity rather than chance. In our study, there are significant and high degrees of heterogeneity for some outcomes. The existing heterogeneity can partly result from different sample sizes, study designs, study times, study scope (nation and region), diagnostic methods, the severity of the disease. We aggregate studies that are different methodologies, but the heterogeneity in the results is still inevitable.

### Methodological Considerations

To our knowledge, this is the first meta-analysis that summarizes the comparison of clinical information on STEMI patients presenting with vs. those presenting without COVID-19 infection. We included multiple studies that were conducted in Asia, Europe, and North America, so that our findings can provide a broad overview of COVID-19 infection in patients with STEMI. However, our study has several limitations. First, the delay time, laboratory values, and length of stay were reported in terms of median values and IQR in many studies, which have been adjusted to means and SDs using the Box-Cox method. Nevertheless, using this method to calculate SDs may entail inaccuracy and make the SDs greater than the mean in some cases, which is an inherent feature of the method (17). Second, the disparity in study size may affect the weighting of the studies and the pooled effect size, which is innate to meta-analyses (53, 54). Third, a high degree of heterogeneity was observed in some outcomes. Due to inadequate information for the included studies, it is difficult to conduct a subgroup analysis to explain the heterogeneity. We performed a sensitivity analysis to assess the reliability of our findings and used the random-effects model when I^2^ statistics were more than 50%. Fourth, we were unable to compare the rate of thrombosis and elective PCI, and the revascularization rate of patients undergoing primary angioplasty between the two groups due to a lack of sufficient data. Future studies are needed to further investigate these outcomes. Finally, our data were limited to in-hospital outcomes. Long-term follow-up is required to explore the association between SARS-CoV-2 infection and poor outcomes in patients with STEMI.

## Conclusion

In patients with STEMI, COVID-19 has had a deep impact on their therapeutic management and clinical outcomes. A longer time from SO-to-FMC and D2B was observed in STEMI patients with COVID-19 in our study. Moreover, patients with STEMI who also had COVID-19 had more severe thrombotic events adverse outcomes. Further studies are required to explore the mechanism of coronary thrombus burden and the optimal treatment for patients with STEMI and COVID-19.

## Data Availability Statement

The original contributions presented in the study are included in the article/supplementary material, further inquiries can be directed to the corresponding author/s.

## Author Contributions

YW, LK, C-WC, SX, and T-HT: conception. YW, LK, C-WC, JX, PY, SX, and T-HT: methodology. YW, LK, JX, PY, and T-HT: analysis. YW, LK, JX, and PY: interpretation and writing. C-WC, SX, and T-HT: supervision. All authors have read and agreed to the published version of the manuscript.

## Conflict of Interest

The authors declare that the research was conducted in the absence of any commercial or financial relationships that could be construed as a potential conflict of interest.

## Publisher's Note

All claims expressed in this article are solely those of the authors and do not necessarily represent those of their affiliated organizations, or those of the publisher, the editors and the reviewers. Any product that may be evaluated in this article, or claim that may be made by its manufacturer, is not guaranteed or endorsed by the publisher.
